# Development and Deployment of DeepSeek‐Based Applications in Healthcare: A Chinese Perspective

**DOI:** 10.1002/hcs2.70068

**Published:** 2026-06-21

**Authors:** Maoxin Lv, Ning Li, Hui Zhang, Chao Liu, Ge Wu, Zheng Zhu, Yao Zhu, Mengchun Gong

**Affiliations:** ^1^ Department of Urology, First Affiliated Hospital Kunming Medical University Kunming China; ^2^ School of Biomedical Engineering Guangdong Medical University Dongguan China; ^3^ GMC Lab, School of Biomedical Engineering, School of Biomedical Engineering Guangdong Medical University Dongguan China; ^4^ Peking Union Medical College Hospital Chinese Academy of Medical Science and Peking Union Medical College Beijing China; ^5^ Digital Health China Technologies Co. Ltd. Beijing China; ^6^ Department of Urology Xijing Hospital of Air Force Military Medical University Xi'an China; ^7^ Department of Urology Fudan University Shanghai Cancer Center Shanghai China; ^8^ Department of Oncology, Shanghai Medical College Fudan University Shanghai China

**Keywords:** application of artificial intelligence in healthcare, artificial intelligence, deepseek model, healthcare resource equity

## Abstract

**Background:**

Artificial intelligence (AI) is already showing enormous potential in the healthcare sector. Generative AI, particularly, is accelerating the sector's digital transformation by delivering intelligent decision support, automated diagnosis, and optimized resource allocation. DeepSeek‐R1, a large‐language model with a strong performance‐to‐cost ratio, has gained popularity as a foundation model in Chinese hospitals. However, the deployment of generative AI remains challenging, and hospitals continue to lack clear guidance on how to select among deployment architectures and how to balance computational demand with cost. Deeper, data‐driven analysis is therefore warranted to inform future roll‐outs.

**Methods:**

This study surveyed AI deployment across Chinese hospitals, with a focus on DeepSeek's potential applications under national policies. Data were collected from the top 20 hospitals, regional centers, and township hospitals via official WeChat platforms. The survey examined deployment strategies, model versions, and platform choices, while keeping in account hospital needs, data resources, and technological‐economic decisions.

**Results:**

The study highlights DeepSeek's impact on diagnostic accuracy, personalized treatment, medical documentation automation, and resource management optimization. Among the 17 surveyed hospitals, 6 hospitals employed detailed model versions, 5 used the 671B model, and 1 used the 32B version. Among 10 hospitals of different levels, 2 selected the 671B, 3 selected the 70B, and 4 selected the 32B model. All hospitals preferred local deployment. Different needs and applications were observed across the studied hospitals.

**Conclusions:**

Selection of the right AI model requires balancing computational power with cost. Larger models offer higher accuracy, but incur higher costs, whereas distilled models suit smaller hospitals with fewer resources. Future development should therefore focus on selecting deployment strategies based on the hospital size while addressing data quality disparities to bridge the regional healthcare gaps. As such, coordination among government, hospitals, and doctors is crucial for supporting smarter healthcare transitions.

## Graphical abstract

Generative Artificial Intelligence based on DeepSeek drives the comprehensive development of healthcare in China, demonstrating potential application across multiple areas.

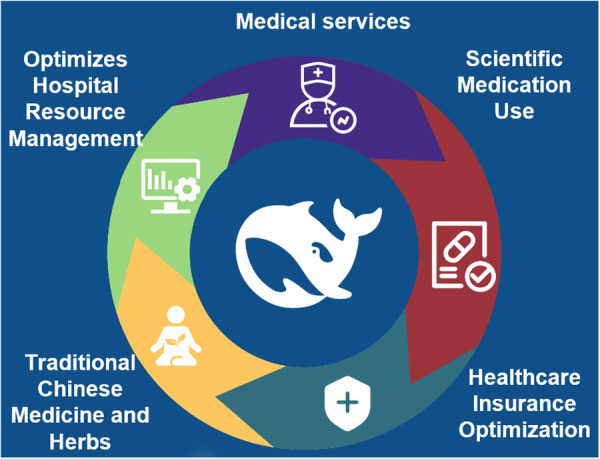

**Abbreviations**
AI: artificial intelligence
GAI: generative AI

## 1 Background

Recent advancements in artificial intelligence (AI) have led to transformative changes in the healthcare vertical, offering opportunities to enhance clinical decision‐making, diagnostic accuracy, and personalized treatment plans [1].

In China, the open‐sourcing of generative AI (GAI) models, such as DeepSeek‐R1, has significantly impacted healthcare, demonstrating performance comparable to that of ChatGPT‐O1, albeit at a lower training cost [2, 3]. DeepSeek has been endorsed by leading hospitals and is being increasingly adopted as the foundational model for large‐scale healthcare deployments.

**Figure 1:**
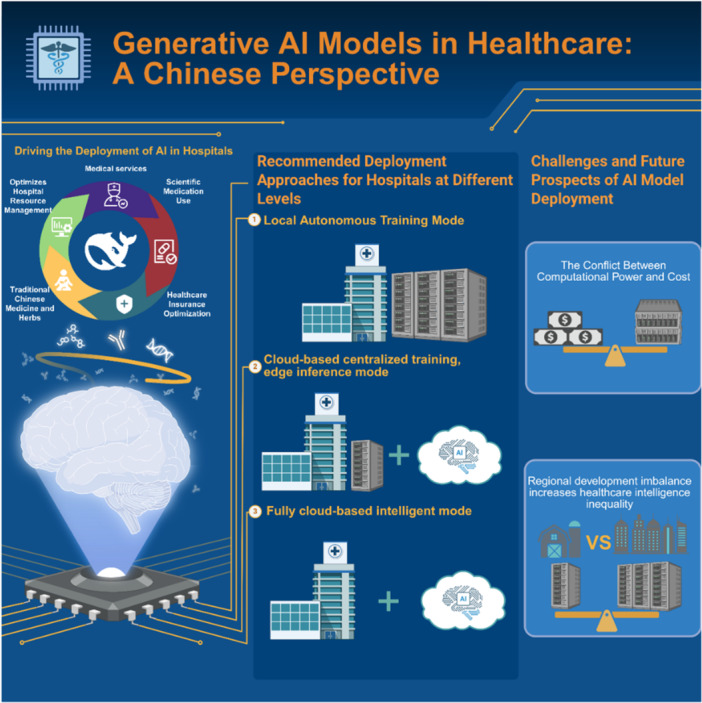
Potential applications of generative artificial intelligence in healthcare deployment, possible deployment methods, and future challenges.

Despite the promise, the implementation of AI in healthcare faces substantial challenges, including the absence of mature deployment solutions and the lack of clarity around the return on investment. The quality of local data and cost constraints contribute to significant variability in AI adoption across hospitals. The current gap in intelligent development raises concerns about the potential disparities in healthcare access and equity. This paper describes the state of DeepSeek‐R1's early open‐source deployment in Chinese hospitals, analyzes its economic, clinical, and social implications, as well as explores the future development trends, offering theoretical support for healthcare decision‐makers in navigating AI integration [4] (Figure 1).

## 2 Methods

This research systematically evaluates DeepSeek deployment across Chinese hospitals, particularly when examining DeepSeek's applications under national policy guidance.

Data collection was conducted between January 20, 2025 (when the DeepSeek‐R1 model became publicly available) and March 20, 2025. The study aimed to assess the current state of AI implementation in Chinese hospitals during the early stages of AI development, while identifying the potential underlying challenges.

A stratified sampling approach was employed to capture a representative range of hospitals. The hospitals were classified into 10 levels in accordance with the guidelines of the Ministry of Health of the People's Republic of China [5]. In the first phase, deployment information was gathered from the top 20 grade hospitals, as per the 2024 Fudan China Hospital Comprehensive Rankings. These 3AAA‐level hospitals represent the pinnacle of medical care in China. In the second phase, data were collected from the initial 10 hospitals to adopt DeepSeek‐R1 across the remaining nine hospital levels. To ensure the accuracy and reliability of the data, we utilized hospitals' official WeChat Accounts as the primary source for information gathering.

This approach allowed the assessment of AI deployment differences under varying resource conditions and provided targeted recommendations for different medical institutions. The following four key dimensions formed the assessment framework: deployment strategies; DeepSeek model version selection; platform selection; and application scenarios.

This framework enabled systematic evaluation of AI deployment status and requirements across different medical institutions, creating a comprehensive understanding of current implementation practices and future development pathways based on empirical evidence.

## 3 Results

### 3.1 DeepSeek‐Based Generative AI Application Scenarios in the Healthcare Sector

In 2024, the General Office of the National Health Commission of the People's Republic of China, along with the National Administration of Traditional Chinese Medicine of the People's Republic of China and the National Disease Control and Prevention Administration of the People's Republic of China, jointly published the “*Reference Guide for AI Application Scenarios in the Healthcare Industry*” [6]. This document outlines four core areas and 84 specific application scenarios, providing a clear blueprint for the intelligent transformation of the healthcare sector. The policy‐driven deployment of DeepSeek‐based GAI in China is expected to play a pivotal role across various sectors [7]. Considering that DeepSeek‐R1's performance is on par with that of ChatGPT‐O1, it has generated significant expectations within the medical field. As a result, there is a growing belief that this model could significantly contribute to the advancement of AI applications in healthcare, addressing diverse challenges and improving efficiency in medical practices.

#### 3.1.1 DeepSeek‐Based Generative AI May Improve the Efficiency of Medical Services

DeepSeek‐based AI may contribute toward optimization of healthcare through intelligent triage, case generation, personalized follow‐up plans [8], and enhanced medical imaging [9, 10, 11, 12]. It improves diagnostic accuracy [11], supports continuous patient monitoring, and generates personalized treatment plans, thereby boosting surgical precision [13, 14]. AI enhances patient experience by reducing the waiting period and increasing appointment accuracy [15, 16], thereby driving the overall hospital efficiency and satisfaction [17, 18, 19, 20, 21].

#### 3.1.2 DeepSeek‐Based GAI May Improve the Science of Medication

GAI offers personalized medication guidance, clinical support, and prescription auditing, which together enhance adherence and reduce errors. It provides tailored drug recommendations, optimizing choices, dosages, and durations, especially for complex cases. AI also monitors medication safety, manages treatment risks, and ensures rational drug selection, which improves the pharmacotherapy outcomes.

#### 3.1.3 GAI May Facilitate Rational Planning of Medical Insurance

GAI automates medical record reviews, improving audit accuracy and efficiency [22]. By analyzing healthcare data for risk assessments, AI enhances underwriting, reduces risks, and optimizes insurance pricing and product design [23]. This approach fosters personalized, flexible plans, strengthens risk control, and drives growth and innovation in the commercial health insurance sector.

#### 3.1.4 DeepSeek‐Based Generative AI Is Likely to Contribute to the Development of Traditional Chinese Medicine

DeepSeek‐based GAI may play the key role in traditional Chinese medicine by generating personalized treatment plans based on patient data, which facilitates meridian health detection and review of herbal prescriptions for safety and efficacy [24, 25, 26]. It also helps practitioners stay updated with the latest research and supports drug development and the design of new formulas [27], which help accelerate the modernization and precision of herbal medicine.

#### 3.1.5 Generative AI is Likely to Revolutionize Resource Management in Hospitals

DeepSeek‐based GAI offers the potential to streamline resource distribution across hospitals, toward improved efficiency and reduced waste [28]. It enhances document quality by providing real‐time feedback, optimizes staff allocation, surgery scheduling, and equipment management [29, 30]. AI also supports economic management by analyzing operational data, aiding decision‐making, and ensuring optimal resource distribution across hospital operations [28].

### 3.2 Performance and Cost of Deploying DeepSeek Model Versions

The primary considerations for hospitals in relation to the deployment and application of DeepSeek‐based GAI are with reference to healthcare, deployment costs, and benefits. The deployment strategy serves as the foundational design toward shaping the future trajectory of hospital automation. A thorough understanding of these aspects is therefore essential.

#### 3.2.1 Cost of Deploying DeepSeek Models

DeepSeek offers a range of performance options, from the full version with 671B parameters to the distilled model with 1.5B parameters. The full version is a complete, large model, featuring stronger inference capabilities and higher accuracy, which is ideal for complex tasks such as deep analysis, long‐text processing, causal reasoning, and complex decision support. In comparison, the distilled model employs knowledge distillation techniques to extract a lighter version from the full model and optimizes resource usage and model size, making it more suitable for resource‐limited environments. However, the distilled model experiences a slight drop in inference capability and performance, which is primarily used for efficient inference and low‐latency applications, albeit it maintains a high level of performance and accuracy. Table 1 presents the hardware configuration requirements and cost estimates for different model versions. The larger parameter versions demand more hardware support, because of which hospitals face higher deployment costs, and the cost of hardware increases exponentially with model size. In medical contexts, 90% of the routine tasks are based on text information processing. It remains uncertain whether extensive high‐end hardware support is necessary in such cases. Nonetheless, optimizing costs while meeting the demands is a critical challenge to address in the initial stages of AI deployment.

**Table 1:** Hardware requirements for different versions of DeepSeek.

Model parameters	CPU (cores)	Memory (GB)	GPU memory (GB)	Storage space (GB)	Estimated cost (CNY/year)
7B	8	32	8 (RTX 3090)	≥ 10	50,000–100,000
32B	16	128	24 (RTX 4090)	≥ 30	100,000–300,000
70B	32	256	40 (A100)	≥ 100	400,000–1,000,000
671B	64	512	160 (8xA100)	≥ 500	≥ 2,000,000

Abbreviations: CNY, Chinese Yuan; CPU, Central Processing Unit; GB, gigabyte; GPU, Graphics Processing Unit.

#### 3.2.2 Deployment Methods for DeepSeek Model

Currently, the three commonly used AI computing power platform deployment models are fully local deployment, cloud‐based centralized training with edge inference, and fully cloud‐based computing power platform.

The fully localized deployment model places all computational infrastructure within the organization, supporting both model training and inference tasks. All data is processed locally, ensuring high security and privacy protection, particularly for sensitive healthcare data. In addition, local deployment allows millisecond‐level response, fulfilling the demands of real‐time tasks and computational power. However, this model requires healthcare institutions to invest significant capital in high‐performance hardware, such as Graphics Processing Unit clusters and large‐capacity storage systems. The annual maintenance costs may exceed one million yuan, and its scalability is limited, which makes it hard to adapt to sudden computational needs.

The hybrid model integrates cloud‐based training with edge inference, reducing hardware demands on edge devices while leveraging cloud computing for complex tasks. Although it enhances efficiency, its performance is heavily dependent on network stability. Edge devices may struggle with demanding tasks, such as multi‐modal tumor segmentation, which could compromise real‐time performance for large‐scale operations.

The fully cloud‐based model outsources all computational resources to cloud providers, thereby reducing initial investment and offering flexible scalability, making it ideal for small or budget‐constrained institutions. This model also provides the advantage of elastic scalability, facilitating adaptation to intermittent large‐scale data analysis needs in clinical settings. However, the ongoing costs may surpass those of local deployment, and potential data privacy concerns necessitate a thorough evaluation of whether cloud services comply with healthcare data protection standards.

### 3.3 Levels of Deployment and Deployment Methods for DeepSeek Model in Hospitals

We have summarized the deployment strategies of 20 hospitals rated A++++ in the Fudan version of the “2024 China Hospital Comprehensive Rankings” (Table 2). Notably, the deployment methods of large tertiary hospitals may not be strongly applicable to most hospitals. We collected and compared the deployment plans of representative regional medical centers and hospitals of different levels from 10 prefecture‐level cities (Table 3). Through examining the existing deployment methods, we analyzed and explored the optimal deployment models.

**Table 2:** DeepSeek deployment status in A++++ hospitals of the 2024 Fudan China Hospital Comprehensive Rankings.

Hospital	DeepSeek version	Deployment method	Features
PLA General Hospital	DeepSeek‐R1	Local deployment	Data must remain within the hospital, combining Ascend hardware and MindIE inference platform, which uses the MaxKB tool to build a local knowledge base
Peking Union Medical College Hospital	DeepSeek‐R1 (671B)	Local deployment	Combines other open‐source models with quantum security to launch “Xiehe Smart Hub”, using Xiehe's experience to create a multi‐scenario AI application integrating healthcare, service, and management
Peking University First Hospital	DeepSeek‐R1 (671B)	Local deployment	Collaborates with Qwen‐Plus for research Q&A, literature reading, paper editing, and intelligent decision support for innovative drug clinical applications
Peking University Third Hospital	DeepSeek‐R1 (Multiple Versions)	Local deployment	Handles everyday lightweight tasks like medical knowledge retrieval and basic diagnostic support
China Medical University First Hospital	DeepSeek‐R1	Local deployment	Uses the Ascend computing platform to complete case discussions, optimize medical records, assist in document generation, and extract medical data to support diagnostic decisions
Shanghai Jiao Tong University School of Medicine Affiliated Renji Hospital	DeepSeek‐R1	Local deployment	Uses MindSpore, Ascend, and AI agents for medical record content quality control, record assistance, case analysis, and literature support
Shanghai Jiao Tong University School of Medicine Affiliated Ruijin‐Hospital	Unknown	Unknown	Co‐developed with the RJH‐Base foundation to create the Ruijin Medical Model Matrix, launching the RuiPath pathology model based on Huawei's full‐stack AI solution
Fudan University Zhongshan Hospital	DeepSeek‐R1 (671B)	Local deployment	For hospital management, clinical diagnosis, scientific teaching, research, and patient services. Based on the cardiovascular knowledge base, the first cardiovascular specialty model‐Guanxin Large model‐was launched
Fudan University Huashan Hospital	DeepSeek‐R1 (671B/70B)	Local deployment	Explores the best cost‐performance ratio through multi‐model configuration
Zhejiang University First Hospital	Unknown	Unknown	Unknown
Zhejiang University Second Hospital	Unknown	Unknown	Unknown
Zhengzhou University First Affiliated Hospital	DeepSeek‐R1	Unknown	Provides innovative services for patients covering pre‐diagnosis, during diagnosis, post‐diagnosis, and the full process, continuously enhancing the patient experience
Tongji Hospital, Tongji Medical College, Huazhong University of Science and Technology	DeepSeek‐R1	Local deployment	Used for medical document support functions
Huazhong University of Science and Technology, Tongji Medical College, Xiehe Hospital	DeepSeek‐R1 (32B)	Local deployment	Deploys large model capabilities to the frontline of diagnosis and treatment, establishing a secure and controllable privatized AI platform
Xiangya Second Hospital, Central South University	Unknown	Unknown	Unknown
Xiangya Hospital, Central South University	DeepSeek‐R1	Local deployment	Aggregates over 2.5 billion medical records, processed through medical logic to create over 10 specialized disease databases
Sun Yat‐sen University First Affiliated Hospital	DeepSeek‐R1 (671B)	Local deployment	Collaborated with “Shenzhou Medical” to create a specialized peritoneal dialysis large model, integrating DHC and DeepSeek dual‐engine architecture with multimodal fusion technology to accurately understand and process complex peritoneal dialysis information
Southern Medical University Nanfang Hospital	DeepSeek‐R1	Local deployment	Intelligent physical examination report interpretation system, fully automated general checkup report generation tool, and localized health management knowledge base
West China Hospital, Sichuan University	Unknown	Local deployment	To realize “AI+diagnosis”, “AI+service,” and “AI+management,” integrates extensive medical data to build a 72‐billion‐parameter fully self‐owned intellectual property “West China Hongyi” medical model
Xijing Hospital	Unknown	Unknown	Unknown

Abbreviations: AI, artificial intelligence; Q&A, question and answer.

**Table 3:** DeepSeek deployment in healthcare institutions at different levels.

Hospital	Hospital level	DeepSeek version	Deployment method	Requirement
Xinqiao Hospital, Army Medical University	A3	DeepSeek‐R1 (70B/32B)	Local Deployment	Medical knowledge Q&A, case analysis support, medical document generation, and daily office tasks
Chongqing University Three Gorges Hospital	A3	DeepSeek‐R1 (70B)	Local Deployment	Disease analysis, report interpretation, and clinical treatment recommendations
The Third Affiliated Hospital of Jinzhou Medical University	A3	DeepSeek‐R1 (32B)	Local Deployment	Document handling, contract review, and quality control analysis
The First Hospital of Hebei Medical University	A3	DeepSeek‐R1 (671B)	Local Deployment	Rapid and precise diagnosis improves medical record writing quality and work‐related applications
Yuxi People's Hospital	A3	DeepSeek‐R1 (32B)	Local Deployment	Optimizing natural language descriptions for image features, image enhancement, and office assistant
Guangdong Provincial Maternal and Child Health Hospital	A3	DeepSeek‐R1 (671B)	Local Deployment	Build a knowledge base, one large inquiry model, multiple small triage intelligent agents covering guidance, diagnosis support, and health management
Beihai People's Hospital	A3	DeepSeek‐R1 (32B)	Local Deployment	Improve document quality, elevate hospital service, and management
Gongyi People's Hospital	B3	DeepSeek‐R1 (32B)	Local Deployment	Medical record management, clinical care, health education, administrative logistics, and patient services
Liuzhou Red Cross Hospital	A2	DeepSeek‐R1 (70B)	Local Deployment	Mobile hospital AI guidance, doctor's assistant, medical record quality control system, OA office system intelligence, and material management
Rongxian Second People's Hospital	B2	DeepSeek‐R1 (70B)	Local Deployment	Intelligent decision‐making support, treatment plan optimization, and medical record quality control enhancement

Abbreviations: AI, artificial intelligence; OA, office automation; Q&A, question and answer.

In the top 20 hospitals, the AI deployment strategies reflect robust resource capabilities. Among the 17 surveyed hospitals, with 6 hospitals providing detailed model versions, 5 used the 671B model and 1 used the 32B version. However, some hospitals, such as Ruijin‐Hospital and West China Hospital, have leveraged their strengths to build independent large models. Among them, some hospitals aiming to integrate AI into frontline clinical care have deployed the 32B distilled model. Crucially, local deployment has become the primary choice for all hospitals, driven partially by policy requirements [31, 32, 33] and partially by the complexity of processing patient information in the desensitization process [34].

Furthermore, other hospitals have exhibited various deployment strategies. In the development of large models, large hospitals pursue medical intelligence matrix integration, whereas other hospitals aim to specialize in a single domain. For example, Peking University First Hospital's AI research, the “Peritoneal Dialysis Large Model,” co‐developed by Sun Yat‐sen University First Hospital and Digital Health China Technologies Co. Ltd., integrates over 60 years of nephrology research data from Sun Yat‐sen's nephrology department [35]. This model combines Digital Health China Technologies Co. Ltd.'s large model AI technology with the DHC+DeepSeek dual‐engine architecture to create a comprehensive “data‐knowledge‐decision” intelligent management system. The AI‐powered obesity model for children and adolescents, launched by the National Children's Medical Center, Shanghai Children's Medical Center, and the company, has been activated to support obesity prevention across hospitals, schools, families, and communities. In the future, it will be expanded across multiple centers, including the National Children's Regional Medical Center [36].

However, research data from regional medical centers and primary hospitals show that the 70B and 32B distilled models are more commonly selected. In the survey of 10 hospitals of different levels, 6 used the 32B version, 3 selected the 70B version, and only 1 deployed the full 671B model.

Overall, the DeepSeek AI models deployed by top‐tier hospitals have larger model parameters and some level of medical tuning. In contrast, the models deployed in hospitals of other levels have smaller model parameters and lack medical‐specific adjustments for several of the deployed models.

The analysis identified two primary factors contributing to the disparities in model deployment between 3AAA hospitals and those of other levels. First, economic considerations were found to be a significant determinant, as 3AAA hospitals benefit from substantial financial resources. Second, differences in the application needs emerged as the key factor. Lower‐level hospitals face challenges such as suboptimal local data quality, which complicates the application of large models for tasks such as diagnostic recommendations and other multimodal applications. In contrast, text‐based administrative tasks, which are common in these hospitals, do not require models with extensive parameters.

These smaller models are well‐suited to daily medical tasks, capable of generating medical records, interpreting reports, and providing basic diagnostic support, with notable advantages in cost control and maintenance. In contrast, although the full version of the large model offers powerful computational capabilities and precision, these advantages may not be essential for several regional and smaller hospitals. Selecting the most suitable version that ensures task completion efficiency while minimizing costs and maintenance complexity is therefore a more prudent choice.

## 4 Discussion

### 4.1 Multiple Advantages Driving the Deepseek Model in Chinese Hospitals

The application of AI technology in healthcare is expanding, covering a wide range of scenarios, from clinical decision support to healthcare operations management. In all these roles, AI is playing an increasingly important role. The introduction of AI offers hospitals the opportunity to improve diagnostic efficiency, optimize resource distribution, and enhance healthcare quality while delivering substantial economic and social benefits to the healthcare industry. AI is also accelerating the intelligent transformation of hospital management and healthcare services.

GAI is a key component of the AI field, leveraging algorithms and model reasoning to learn and generate logically coherent new content. Its greatest strengths lie in its creativity and flexibility. Unlike conventional AI, which relies on explicit rules and supervised learning, GAI can create new content, such as text, images, and drug molecules, based on the existing data, thereby offering substantial innovative potential. The application of GAI in the medical field is particularly extensive, with enormous potential for deployment within hospitals.

As a leading representative of GAI in China, DeepSeek offers significant advantages in healthcare applications. Its highly efficient architecture design optimizes computational resources through multi‐head attention and mixture‐of‐experts techniques, thereby enhancing the ability to process large‐scale medical data. In addition, FP8 (8‐bit floating point) mixed‐precision training and distributed optimization enhance training efficiency and reduce the computational costs [37]. Through reinforcement learning, DeepSeek can independently enhance its inference ability and manage complex healthcare decision‐making tasks. Deep causal reasoning strengthens the understanding of disease causes, enhances the model's interpretability, and helps medical staff trust and utilize AI‐driven decisions more effectively. Furthermore, DeepSeek supports real‐time learning and adaptation, ensuring it stays up‐to‐date with the latest clinical data. The key advantage of DeepSeek is its open‐source nature, which allows users to deploy it at a lower cost relative to GPT‐4. These benefits have driven the rapid deployment of DeepSeek in hospitals across China.

Presently, large medical models have rapidly advanced, with over 50 large models targeting the medical field already developed both domestically and internationally, and this number is continuously increasing. These models, however, have already been actively implemented in real‐world hospital settings. According to the 2023 survey data from the American Hospital Association [38], 65% of all hospitals report using electronic health records (EHR) that include integrated AI or predictive models. The most common sources of predictive models are EHR developers (79%), third‐party developers (59%), and in‐house developed models (54%). Common applications include predicting the health trajectory of hospitalized patients, identifying high‐risk outpatient patients, and aiding in scheduling. In China, the emergence of the domestic AI large model DeepSeek‐R1 has rapidly accelerated the adoption of intelligent technologies. Owing to its multiple algorithmic advantages, DeepSeek has become the preferred choice for several hospitals in deploying large‐scale models.

### 4.2 Challenges and Future Prospects of AI Model Deployment

#### 4.2.1 The Conflict Between Computational Power and Cost Is Crucial for Deployment

Although the full version of the large model exhibits significant advantages in processing complex tasks owing to its powerful computing capacity and high precision, its massive hardware requirements and the high operational costs present significant budget challenges for several resource‐constrained hospitals. Especially in the daily work of many hospitals, tasks such as assisting with medical record generation, interpreting reports, and providing basic diagnostic support do not demand high computational power. Therefore, selecting the right model version is more critical than simply pursuing the most powerful “full” version in terms of computational capacity.

Therefore, when selecting a DeepSeek model for deployment, hospitals must consider their specific needs, data resources, budget, and maintenance capabilities, rather than simply opting for the most computationally powerful full version. Properly configuring the model can enhance medical efficiency, optimize resource utilization, and drive the efficient use of AI in healthcare. Analyzing from a technical‐economic standpoint, although the full version excels in complex diagnostic scenarios, its high deployment cost imposes a significant burden on most healthcare institutions. In comparison, the annual cost of the 32B version can be kept under 300,000 yuan. Ongoing practices have demonstrated that, for routine medical tasks, such as medical record quality control and order verification, the distilled model's performance is sufficient to meet clinical requirements. Given the current circumstances, distilled large models may become the mainstream choice for regular hospitals.

A suitable model version can maintain work efficiency and accuracy while lowering the costs and easing the maintenance‐related challenges. Hospitals should also consider the availability of data, technical adaptability, and localization needs when selecting an AI model for use. Depending on their size, business requirements, and technical conditions, different hospitals may need different deployment models. When selecting a model, hospitals should therefore conduct a comprehensive evaluation from different perspectives. Clinical needs should be classified, distinguishing complex tasks such as difficult case consultations from routine medical documentation; data resources showed be evaluated, including data size, quality, and annotation level; total ownership costs should be calculated, short‐term investments should be balanced with long‐term returns; and technical support capabilities should be assessed to select an appropriate version based on these factors. Large hospitals, with rich medical data and resources, are better suited for local deployment, utilizing deep learning and multimodal technologies to tackle complex tasks. In contrast, local and community hospitals with limited data resources should prioritize model version adaptability to ensure the technology can efficiently support everyday medical tasks.

#### 4.2.2 Optimizing Deployment Approaches to Promote Balanced Development of Healthcare Resources

The future of medical AI development should focus on promoting balanced healthcare development across regions, rather than widening the technological gap between local and top‐tier hospitals. To ensure the widespread adoption and effective use of AI technology, hospitals should select suitable deployment methods based on their specific needs and conditions, avoiding the formation of technological “silos.”

For example, decision‐making by intelligent systems is typically based on multimodal outcomes. A key characteristic is that these leading hospitals deploy large medical models, possessing rich clinical data and resources, which enable them to construct large model platforms integrating local medical information and multimodal data fusion. Patient data is complex and multimodal. AI applications in the medical field should therefore be based on multimodal data and multi‐omics, and offer appropriate responses considering the various time and spatial dimensions. Large hospitals, with several years of accumulated medical experience, generally have extensive knowledge bases. The exceptional data samples provided by AI local deployments in these hospitals place them at the forefront of AI healthcare resource deployment in China.

In the U.S. healthcare system, notable differences exist in the use and evaluation of clinical predictive models. For instance, hospitals integrated into the health system are more likely to use and assess AI models, whereas smaller rural and critical access hospitals are less likely to adopt these technologies [39]. The digital divide in healthcare implies that hospitals with more resources are better positioned to evaluate AI tools, potentially putting under‐resourced hospitals at a disadvantage [38].

Thus, through appropriate technological deployment and resource allocation, AI can become a tool to bridge the gap between hospitals of different levels, assisting local hospitals in improving their service capabilities, optimizing healthcare resource distribution, and further advancing the intelligent and sustainable development of the healthcare industry. Table 4 summarizes the recommended deployment approaches for hospitals at different levels, based on the current DeepSeek deployment status. Leading hospitals and regional healthcare centers can continue to focus on local large model training while developing multimodal intelligent systems for complex scenarios. Secondary and tertiary hospitals or community healthcare facilities can utilize cloud computing power and centralized training platforms to reduce the initial investment, ensuring efficient support for daily diagnostic needs, thereby enhancing regional healthcare service capabilities. This approach allows the application of AI technology across healthcare scenarios, while helping local hospitals better utilize their limited resources, providing patients with higher‐quality and more accessible medical services [40].

**Table 4:** Recommended deployment approaches for hospitals at different Levels.

Platform model	Possess data value	Security requirements	Information technology investment	Recommended hospital level
Local autonomous training mode	High‐value datasets	Data does not leave the hospital	Significant financial and data investment to create a multimodal intelligent matrix	Top‐tier tertiary hospitals, regional healthcare centers
Cloud‐based centralized training, edge inference mode	Limited data quality at hospitals	Data leaves the hospital, but does not go outside the industry data center	An initial high investment in centralized training, with later cost reduction	Regional healthcare centers, secondary and tertiary hospitals
Fully cloud‐based intelligent mode	Poor data quality in hospitals	Data needs to be transferred out of the hospital	Low investment requirement, simple maintenance	Secondary hospitals, community healthcare centers

As illustrated in Figure 1, the powerful capabilities of DeepSeek and its potential application scenarios have generated considerable optimism, albeit the selection of an appropriate deployment framework remains a challenging decision. Currently, there are no outstanding tools to effectively measure the direct relationship between costs and returns, which complicates the decision‐making process. The rapid development of AI products, coupled with the constraints imposed by existing hospital data conditions, has further complicated the balance between investment costs and expected returns. As such, the deployment of AI in hospitals following the open‐source release of the DeepSeek‐R1 model represents a bold and risky endeavor.

From a long‐term usage perspective, local deployment initially incurs high costs but incurs lower maintenance expenses. In contrast, remote deployment emerges as the most expensive operational model. Considering that 3AAA hospitals are at the forefront of medical development in China, they need to invest in local models that can be trained on a secure data foundation for the advancement of the development of medical AI. Once these models are trained, they can be disseminated to lower‐tier hospitals.

Furthermore, for lower‐level regional hospitals, the deployment of DeepSeek presents a risky choice. Owing to the limitations of local data quality and the fact that the current DeepSeek model has not been fine‐tuned for medical scenarios, its application may not fully meet the needs of these institutions. It may be wiser to wait for more mature medical AI products to develop, as they are more likely to deliver higher returns while reducing the risk of worsening healthcare disparities.

The future development of medical AI should help establish a flexible technological ecosystem. It is therefore suggested to adopt the “base model integrated with domain adaptation layer.” Moreover, the same model should be able to dynamically adjust its computation scale, such as supporting elastic scaling from 32B to 671B. Simultaneously, model effectiveness evaluation standards should be established. Moreover, industry regulators should promote the creation of a healthcare AI resource‐sharing platform, enabling knowledge collaboration through regional joint learning and other initiatives. This model could bring the performance of regional medical center models closer to that of top‐tier hospitals [41]. Literature suggests that transfer learning performs best in disease diagnosis and generalization. Coordination from leading hospitals to economically disadvantaged regions is feasible [42]. Therefore, only through this systematic resource optimization path can the promotion of balanced development of healthcare resources by AI technology be truly achieved.

## 5 Conclusions

DeepSeek‐based GAI is transforming China's healthcare system, enhancing the efficiency, accuracy, and cost‐effectiveness of medical operations. DeepSeek has proven crucial in supporting a variety of healthcare applications. By leveraging GAI for tasks such as diagnostic assistance, medical documentation generation, and personalized treatment planning, DeepSeek offers significant benefits, particularly for hospitals aiming to improve service quality.

However, for most hospitals, the cost‐effectiveness of DeepSeek deployment, which strikes the right balance between computational power and cost, is a key consideration. Although large, high‐performance models like DeepSeek‐R1 offer robust diagnostic capabilities, smaller distilled versions provide more practical options for resource‐constrained institutions. Moreover, the choice between local, cloud‐based, or hybrid deployment models, such as cloud‐based centralized training combined with edge inference, significantly influences both deployment strategies and the future development of hospitals.

DeepSeek‐based GAI thus has the potential to bridge technological gaps between hospitals, emphasizing the importance of a balanced and strategic approach toward AI integration in the healthcare segment. It is therefore important to remain mindful of the healthcare resource disparities resulting from differences in data quality.

## Author Contributions

**Maoxin Lv:** writing – original draft (lead); data curation (lead); writing – review and editing; visualization (lead). **Ning Li:** conceptualization (lead); data curation (lead); supervision (lead), validation (lead); writing – original draft (lead); writing – review and editing (lead). **Chao Liu:** writing – original draft (supporting). **Ge Wu:** writing – original draft (supporting). **Hui Zhang:** writing – original draft (supporting), funding acquisition (supporting). **Zheng Zhu:** writing – original draft (supporting). **Yao Zhu:** conceptualization (lead); data curation (lead); supervision (lead), validation (lead); writing – original draft (lead); writing – review and editing (lead). **Mengchun Gong:** writing – original draft (lead); data curation(lead); writing – review and editing; visualization (lead).

## Ethics Statement

The authors have nothing to report.

## Consent

The authors have nothing to report.

## Conflicts of Interest

Chao Liu and Ge Wu are staff of Digital Health China Technologies Co. Ltd. The remaining authors declare no conflicts of interest.

## Data Availability

Data sharing not applicable to this article as no datasets were generated or analyzed during the current study.
